# Effects of the Chiral Fungicides Metalaxyl and Metalaxyl-M on the Earthworm *Eisenia fetida* as Determined by ^1^H-NMR-Based Untargeted Metabolomics

**DOI:** 10.3390/molecules24071293

**Published:** 2019-04-02

**Authors:** Renke Zhang, Zhiqiang Zhou

**Affiliations:** Beijing Advanced Innovation Center for Food Nutrition and Human Health, Department of Applied Chemistry, China Agricultural University, Beijing 100193, China; zhang_ink@cau.edu.cn

**Keywords:** metabolomics, metalaxyl, earthworm, enantiospecific effects

## Abstract

Although metalaxyl and metalaxyl-M are widely used fungicides, very little is known about their subacute and enantiospecific effects on the earthworm metabolome. In this study, *Eisenia fetida* were exposed to metalaxyl and metalaxyl-M at three concentrations (0.5, 5 and 50 mg/kg) for seven days. ^1^H nuclear magnetic resonance (^1^H-NMR)-based untargeted metabolomics showed that metalaxyl and metalaxyl-M exposure disturbed earthworms’ metabolism at all three concentrations. Endogenous metabolites, such as succinate, arginine, aspartate, urea, asparagine, alanine, trimethylamine, taurine, cysteine, serine, threonine, histidine, lysine, glucose, choline, carnitine, citric acid, alpha-ketoisovaleric acid, fumaric acid and so on, were significantly changed. These results indicate that metalaxyl and metalaxyl-M produce different, enantiospecific disturbances in the earthworm metabolism, particularly in the tricarboxylic acid (TCA) and urea cycles. The application of untargeted metabolomics thus provides more information for evaluating the toxic risks of metalaxyl and metalaxyl-M.

## 1. Introduction

Metalaxyl and metalaxyl-M are the world’s most widely used acetylalanine fungicides; they inhibit the synthesis of ribosomal RNA in mycelium. Metalaxyl proper is a racemic mixture [1], while metalaxyl-M is solely (97%) the R-enantiomer [2]. Some studies indicate that the two enantiomers have different effects on the metabolomes of rats [3,4] and human cells [5]. Toxicological evaluations on earthworms found LC50 values for metalaxyl and metalaxyl-M of 0.022 and 0.052 mg cm^−2^, respectively [1], but the underlying mechanism of this toxicity and the lower toxicity of the R-enantiomer is not well understood.

This is a potentially important issue because nearly 80% of the soil animal biomass consists of earthworms, which are key members of the soil ecosystem because of their important role in soil development and maintenance [6]. Earthworms can serve as model organisms to detect soil contamination because they readily ingest pollutants or absorb them through their skin [7,8]. A major goal of ecotoxicology studies is thus to determine the impact of exposure on the behavior, reproduction, growth, and survival of the earthworms. Unfortunately, such studies are sometimes limited in the scope of the pollutant concentrations studied, even though in practice the range of relevant concentrations can be quite wide, due to processes such as pesticide runoff into non-agricultural areas [9]. It is still a challenge to determine the effects of pollutants on target organisms such as earthworms at the low concentrations that can be encountered in the field.

Metabolomics is potentially a powerful tool to accomplish this purpose; it has been used to elucidate the toxic effects of such substances as metals [10], nanoparticles [11], and pharmaceuticals [12]. In particular, metabolomic studies using ^1^H nuclear magnetic resonance (NMR) have proven effective in evaluating the response of organisms to pesticide exposure [13,14,15,16,17,18,19,20], and have become a widely used tool for the study of the effects of soil contaminants on earthworms [21,22,23,24,25]. This method is fast and can determine quantitative changes in the levels of metabolites, such as amino acids and sugars, provoked by an external stressor [26,27,28,29,30]. Being an untargeted technique, such an analysis can be reproduced accurately across different laboratories [31,32,33,34,35,36], providing insights into the reaction of the organism to an external source of stress, its molecular endpoints in terms of changes in metabolite levels, and in depicting the mode of toxicity of the contaminants [37].

The earthworm *Eisenia fetida* was exposed to metalaxyl and metalaxyl-M at three different concentrations for seven days in artificial soil, in order to determine the metabolomic impact of these two fungicides. The concentrations used were 0.5, 5, and 50 mg/kg; these were chosen based on the concentration of fungicide applied in the field and the maximum residue limit (MRL). The main objective was to observe changes in metabolites provoked by metalaxyl and metalaxyl-M exposure, and to evaluate the potential mode of action of metalaxyl and metalaxyl-M in earthworms. Principal component analysis (PCA) was used to provide a quick overview of shifts in metabolites among different earthworm groups. Partial least squares-discriminant analysis (PLS-DA) was then applied for a deeper analysis of the metabolomic data.

## 2. Results and Discussion

### 2.1. Earthworm Body Weight

The control (0.22 ± 0.05 g) and treated groups (0.20 ± 0.03 g) average body weights did not significantly change after 7 days of exposure at all three concentrations tested (Figure S1). No obvious injury or toxic symptoms were observed.

### 2.2. H NMR Spectroscopic Analysis and Multivariate Data Analysis

**Figure 1:**
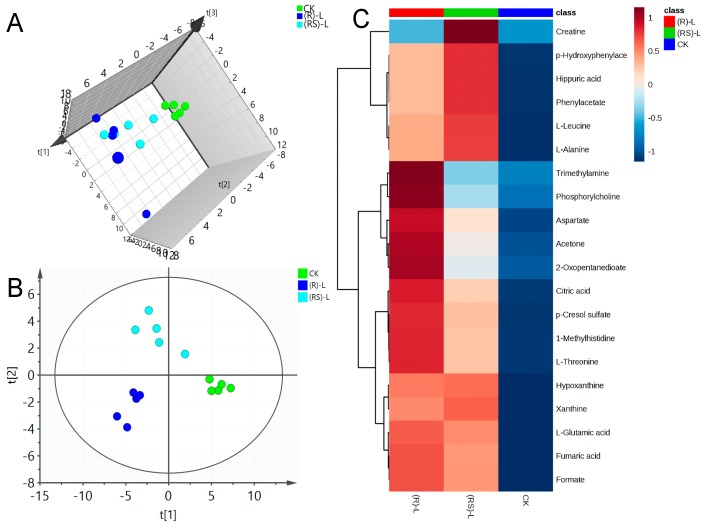
(**A**) Plots for the principal component analysis (PCA) score, (**B**) Plots for the partial least squares-discriminant analysis (PLS-DA) score and (**C**) heat map of earthworm metabolites after exposure to low (0.5 mg/kg) concentration of metalaxyl or metalaxyl-M. (RS)-L: 0.5 mg/kg Metalaxyl, (R)-L: 0.5 mg/kg Metalaxyl-M.

**Figure 2:**
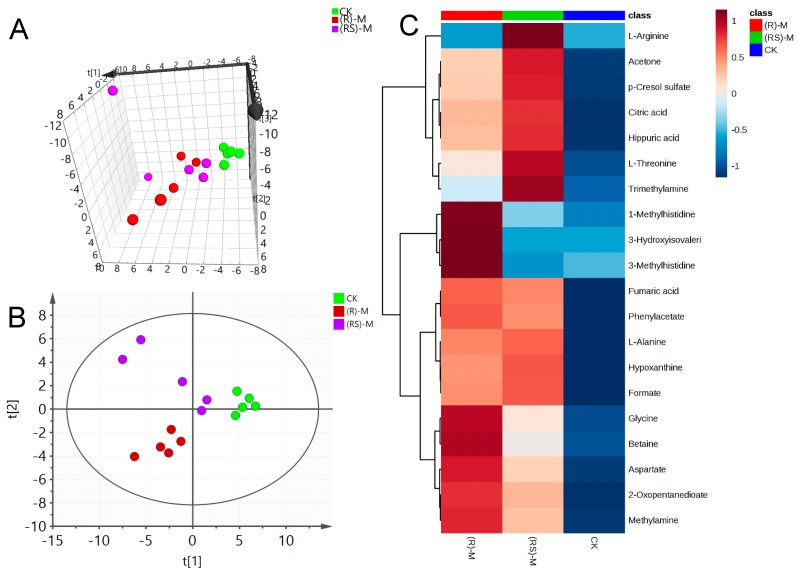
(**A**) Plots for PCA score, (**B**) Plots for PLS-DA score and (**C**) heat map of earthworm metabolites after exposure to medium (5.0 mg/kg) concentration of metalaxyl or metalaxyl-M. (RS)-M: 5.0 mg/kg Metalaxyl, (R)-M: 5.0 mg/kg Metalaxyl-M.

**Figure 3:**
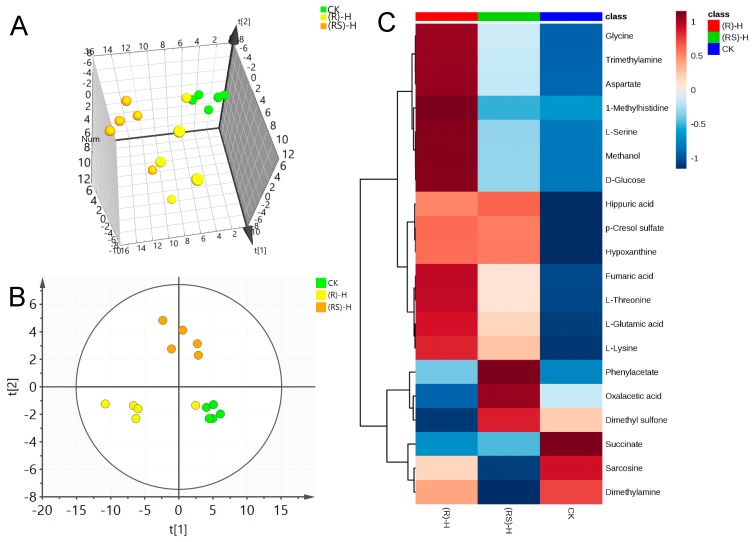
(**A**) Plots for PCA score, (**B**) Plots for PLS-DA score and (**C**) heat map of earthworm metabolites after exposure to high (50 mg/kg) concentration of metalaxyl or metalaxyl-M. (RS)-H: 50 mg/kg Metalaxyl, (R)-H: 50 mg/kg Metalaxyl-M.

A representative 600 MHz 1H-NMR spectrum of a quality control (QC) earthworm sample can be seen in Figure S2. NMR data sets for metalaxyl-exposed earthworms were first analyzed via PCA to determine global changes in metabolites. Being an unsupervised multivariate data analysis technique, PCA is frequently used to envision grouping trends and data outliers. Figure 1A, Figure 2A, and Figure 3A show the principal components PC1, PC2, and PC3; these reveal significant differences between treated and control groups at all three concentrations. This indicates a substantial perturbation of the earthworm metabolome after seven days’ exposure to metalaxyl and metalaxyl-M. In addition, at all three concentrations (0.5, 5.0 and 50 mg/kg) the alterations in metabolome induced by metalaxyl and metalaxyl-M were clearly different, indicating an enantiospecific effect. Moreover, the metabolome change ascribed to three separate fungicide concentrations also varied, as observed when the earthworm treated with Metalaxyl-M at three concentrations were clearly separated on 3D-PCA (Figure S3).

PLS-DA was applied to the NMR data to identify the metabolites responsible for the differences seen in the PCA analysis. As seen in Figure 1B, Figure 2B and Figure 3B, respectively, PLS-DA plots for the treated and the control groups were clearly different. Moreover, the plots for the metalaxyl- and metalaxyl-M-treated groups were also clearly different, which once again indicated an enantiospecific effect. To determine which metabolites were affected by the two fungicides, PLS-DA analysis was used to compare treated groups with the control group. Each independent variable has a key parameter derived from the PLS-DA mode, called the variable importance in the project (VIP) value, which, when high, holds greater relevance is in classification. Thus, each peak’s VIP value was determined to discern its role in the classification.

### 2.3. The Changed Metabolites

**Figure 4:**
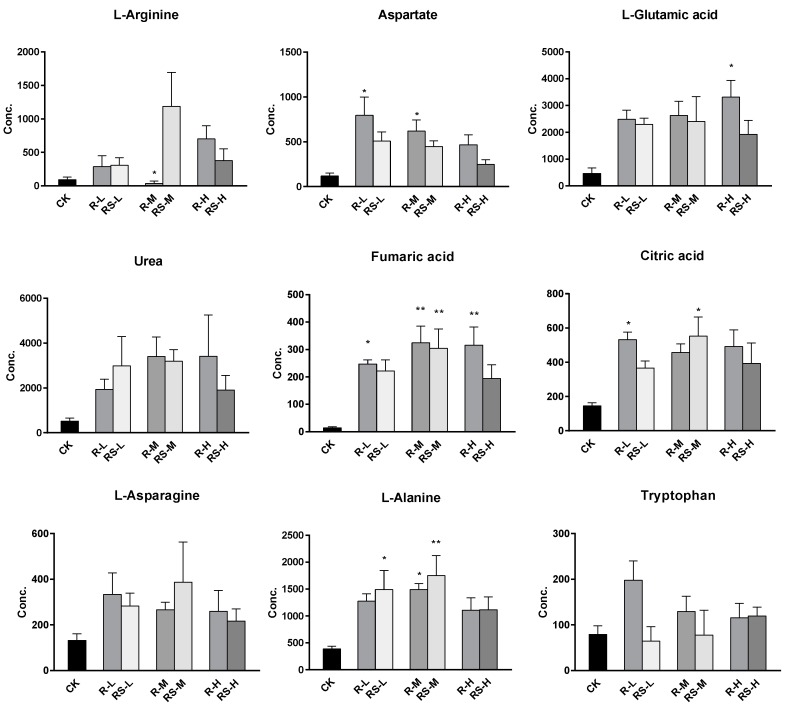
The concentration shifts of some metabolites after exposure to metalaxyl and metalaxyl-M. RS-L: 0.5 mg/kg Metalaxyl, R-L: 0.5 mg/kg Metalaxyl-M. RS-M: 5.0 mg/kg Metalaxyl, R-M: 5.0 mg/kg Metalaxyl-M. RS-H: 50 mg/kg Metalaxyl, R-H: 50 mg/kg Metalaxyl-M.

A total of 51 metabolites whose concentrations were changed by fungicide exposure were finally identified on the basis of Student’s *t*-test (*p* < 0.1) and VIP threshold (VIP >1). The metabolites whose levels were notably altered were carbohydrates, amino acids, phospholipids, nucleotides, fatty acids, indoles, and so on. The groups treated with metalaxyl- and metalaxyl-M at a low concentration (0.5 mg/kg) showed changes in 39 and 33 endogenous metabolites, respectively. Groups treated at a medium concentration (5.0 mg/kg) showed changes in 38 and 35 endogenous metabolites, respectively, while groups treated at a high concentration (50 mg/kg) showed changes in 35 and 33 endogenous metabolites, respectively. These results indicate that metalaxyl-M and metalaxyl have enantiospecific effects on the metabolome. In addition, metabolites like aspartate and arginine—two key metabolites in urea cycle—were altered after 5 mg/kg metalaxyl-M and metalaxyl exposure (Figure 4).

OPLS-DA (orthogonal partial least squares- discriminant analysis) was also used as a multivariate data analysis method; it revealed clear differences in the effects of metalaxyl and metalaxyl-M at all three concentrations tested (Figure S4).

### 2.4. Changes in the TCA and Urea Cycles

**Figure 5:**
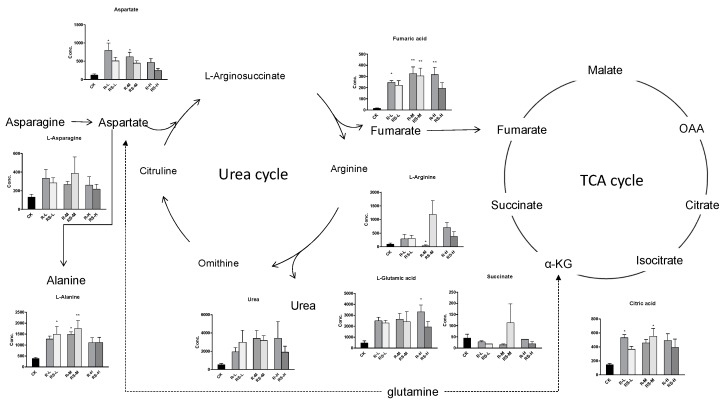
Disturbance of the urea and tricarboxylic acid (TCA) cycle metabolic pathway after metalaxyl and metalaxyl-M treatment.

Noteworthy changes were observed in levels of the tricarboxylic acid (TCA) and urea cycle intermediates (Figure 4). Levels of aspartate and arginine were significantly changed after exposure (Figure 5). Moreover, the 5.0 mg/kg metalaxyl-treated group had a higher concentration of arginine than that in the metalaxyl-M group. Arginine is a precursor for the synthesis of nitric oxide (NO) and plays key roles in several biological processes such as wound healing, cell division, the release of hormones, ammonia excretion, and in the immune system. The ratios of aspartate to arginine showed that the conversion of aspartate to arginine was inhibited at low concentrations of metalaxyl. The aspartate-to-arginine ratio for the metalaxyl-M-treated groups was lower than that for the metalaxyl-treated groups at all three concentrations. At low concentrations, metalaxyl-M was seen to have a greater impact on the urea cycle than metalaxyl, as shown in Figure S5.

Our studies indicate that racemic metalaxyl and R-enantiomeric metalaxyl-M had different impacts on earthworms: Specifically, metalaxyl-M up-regulated the activity of the urea cycle.

## 3. Materials and Methods

### 3.1. Soil Spiking

*E. fetida* were provided by the San Huan Worm Factory (Beijing, China.). The earthworms were raised in OECD (Organisation for Economic Co-operation and Development) artificial soil, containing 67% moisture at roughly 25 °C. The earthworms were maintained in these conditions for several months before their use in metabolomic experiments, in order to minimize the effects of other environmental factors and diet. Metalaxyl (race-metalaxyl) and metalaxyl-M (r-metalaxyl) standards, both of purity ≥98.0% were provided by the Institute for Control of Agrochemicals, China Ministry of Agriculture (Beijing, China).

The earthworm acute toxicity test protocol described by OECD (1984) was followed to prepare the artificial soil, using 10% peat moss (Pindstrup Mosebrug, Ryomgaard, Denmark), 20% kaolin clay and 70% sand. The artificial soil (100 g, dry weight) was then placed in each of seven jars of clear glass and 4 L capacity. Ten mL of metalaxyl or metalaxyl-M were prepared at three separate concentrations (50, 500 and 5000 mg/L) in HPLC grade acetone (Fisher Scientific, Waltham, MA, USA) and used for spiking the six jars. The last glass jar was spiked with only 10 mL of acetone for use as the unexposed control. All the acetone was allowed to evaporate by placing jars for 24 h in the fume hood. Then 900 g of soil was added to each of the jars with spiked soil and mixed thoroughly to achieve a total concentration of 0.5, 5.0 and 50.0 mg/kg (dry weight) of metalaxyl or metalaxyl-M for the pesticide-exposure experiments. Based on the OECD guidelines (1984) deionized water was used to adjust all the soils to 35% moisture content of soil dry weight (OECD, 1984).

### 3.2. Metalaxyl Exposure and Tissue Extraction

Ten mature earthworms with a mean weight of 0.45 ± 0.05 g and a visible clitellum were added to the jars containing each of the seven total types of treated or control soil. No significant difference was observed in the average mass of worms used for the metalaxyl, metalaxyl-M and control groups prior to exposure. In accordance with OECD soil exposure test guidelines, earthworms were maintained in jars at 21 °C for 7 days in natural light. They were then removed, kept on moisturized filter paper, and depurated to remove any surplus soil in their gut. After depuration for 48 h, liquid nitrogen was used to flash freeze the earthworms, which were lyophilized and kept at −20 °C until they were used for the preparation of NMR the samples. Five replicates for the control and for each of the six test groups of exposed worms were used.

For metabolomic studies, the tissue samples of the lyophilized earthworm were put into 2 mL centrifuge tubes. The tubes were then homogenized using the MM 400 Mixer Mills (Retsch, Haan, Germany) with one stainless-steel ball (5 mm wide) in each tube. The extraction of homogenized earthworm tissue was done in 1.2 mL of 0.2 M PBS, in which, the internal calibrant was 10 mg/L of 97% pure 2,2-dimethyl-2-silapentane-5-sulfonate sodium salt (DSS; Sigma Aldrich, St. Louis, MO, USA). The buffer was prepared in 99% pure D2O from Cambridge Isotope Laboratories (Tewksbury, MA, USA). To enable the extraction, the sample was vigorously mixed using a vortex for 30 s, sonicated for 15 min, and then centrifuged for 20 min at 14,000 rpm (21,000× *g*) and 4 °C. The supernatant was isolated and shifted into a fresh centrifuge tube of 1.5 mL capacity. This was replicated two times to eliminate any particle suspension. Finally, the ^1^H-NMR analysis was done by transferring the resulting supernatant into NMR tubes supplied by Norell of 5 mm capacity. From each of all the 35 samples, 40 µL was taken and pooled them as a QC sample.

### 3.3. 1H NMR Spectroscopy

An AVANCEIII600 spectrometer (BRUKER, Billerica, MA, USA) was used to record the NMR spectra of the samples at 600 MHz and 298 K. A 1D NOESY pulse sequence with 20 ppm spectral width was used to acquire all 1D ^1^H-NMR spectra. In the relaxation delay period, the resonance of water was preferentially presaturated. For each spectrum, a 4 s relaxation delay, 2.66 s acquisition time, and 0.01 s mixing time were used to collect 128 transient scans in 64 K data points. Then, using 0.3 Hz line broadening and zero filling to 128 k points, the spectra were automatically Fourier transformed.

All the spectra were processed using the following method. The baseline and phases were manually corrected using MestReNova (version 9.0.1., Mestrelab, Santiago, Spain). The spectral region δ 0.5–9.0 was segmented over a series of 0.04 ppm integral regions. The region near the water resonance (δ 6.1−4.7) was excluded.

Metabolites were identified using the online Human Metabolome Database (HMDB). The biological pathway analysis was based on the Kyoto Encyclopedia of Genes and Genomes (KEGG) pathway database.

### 3.4. Statistical Analysis

The software package SIMCA-P V11.0 (Umetrics, Umeå, Sweden) was used for multivariate data analysis. To generate a group clustering overview and to search for potential outliers, PCA was carried out on the ^1^H-NMR datasets. The metabolites that altered significantly on ^1^H-NMR datasets driven group clustering were explored by the PLS-DA. A Student’s *t*-test was used to discern the dissimilarities in the metabolomes of metalaxyl- and metalaxyl-M-treated earthworms and the control samples. A Student’s *t*-test was applied to identify the altered metabolites (*p* < 0.05 and VIP score > 1.0)

## 4. Conclusions

Metalaxyl and metalaxyl-M were not significantly toxic to earthworms. Few metabolic processes, which might provide the basis for more targeted investigation, were found to be disrupted by metalaxyl exposure. Metalaxyl and metalaxyl-M have different enantiospecific effects and produced different disturbances in the earthworms’ metabolism, particularly in the TCA and urea cycles.

The aspartate levels in the 5 mg/kg metalaxyl-M-treated group were higher than those in the equivalent metalaxyl-treated group. Arginine, one of the downstream metabolites of aspartate in the urea cycle, was seen to have a lower concentration in the 5 mg/kg metalaxyl-M-treated group as compared to the 5 mg/kg metalaxyl-treated group. This implies that metalaxyl has a stronger impact on the urea cycle. The urea cycle is involved in detoxification, and this may be why metalaxyl-M is less toxic to earthworms than metalaxyl [1]. Even as metalaxyl is replaced by metalaxyl-M in many countries, racemic metalaxyl is still one of the most widely used fungicides in the world. Based on this study, the S-enantiomer of metalaxyl should be subjected to further metabolomic and toxicological study.

## Supplementary Materials

The following are available online, Figure S1: Body weight of earthworms before and after exposure, Figure S2: NMR spectrum from quality control group. a leucine, b valine, c 3-hydoxybutyrate, d methylmalonate, e lactate, f alanine, g acetate, h succinate, i citrate, j trimethylamine, k creatine, l taurine, m glycine, n phenylalanine, Figure S3: PCA of metalaxyl and metalaxyl-M at three concentration levels, Figure S4: OPLS-DA (orthogonal partial least squares- discriminant analysis) of metalaxyl and metalaxyl-M at three concentration levels, Figure S5: Disturbance of arginine biosynthesis metabolic pathway after metalaxyl and metalaxyl-M treatment. and Table S1: The fold changes and VIPs of some metabolites after metalaxyl and metalaxyl-M exposed title.
