# Supplementary material for: Effects of the Chiral Fungicides Metalaxyl and Metalaxyl-M on the Earthworm Eisenia fetida as Determined by 1H-NMR-Based Untargeted Metabolomics
Source: Molecules. 2019 Apr 2;24(7):1293. doi: 10.3390/molecules24071293 (PMC6479362; doi:10.3390/molecules24071293)
Supplement: Supplementary file 1 [file molecules-24-01293-s001.pdf]

molecules

Article

Effects of the Chiral Fungicides Metalaxyl and Metalaxyl-M on the Earthworm *Eisenia fetida* as Determined by ^{1}H-NMR Based Untargeted Metabolomics

Renke Zhang [1] and Zhiqiang Zhou [1]*

[1] Beijing Advanced Innovation Center for Food Nutrition and Human Health, Department of Applied Chemistry, China Agricultural University, Beijing 100193, China

* Correspondence: zqzhou@cau.edu.cn; Tel.: +86-10-62733089

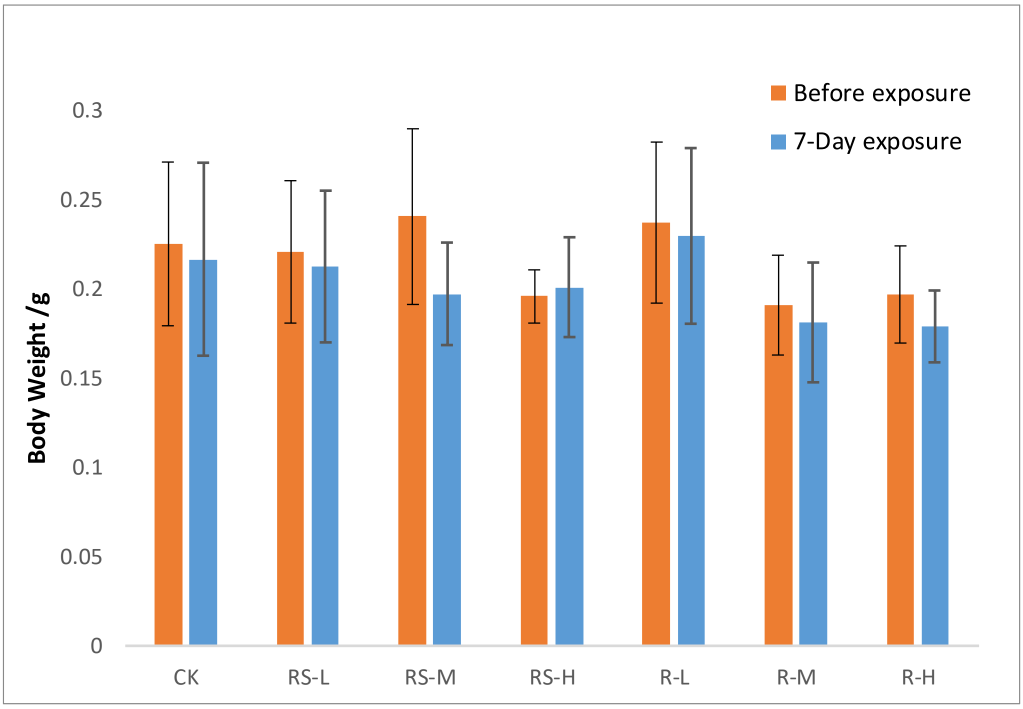

Figure S1. Body weight of earthworms before and after exposure. RS-L: 0.5mg/kg metalaxyl, R-L: 0.5mg/kg metalaxyl-M. RS-M: 5.0mg/kg metalaxyl, R-M: 5.0mg/kg metalaxyl-M. RS-H: 50mg/kg metalaxyl, R-H: 50mg/kg metalaxyl-M.

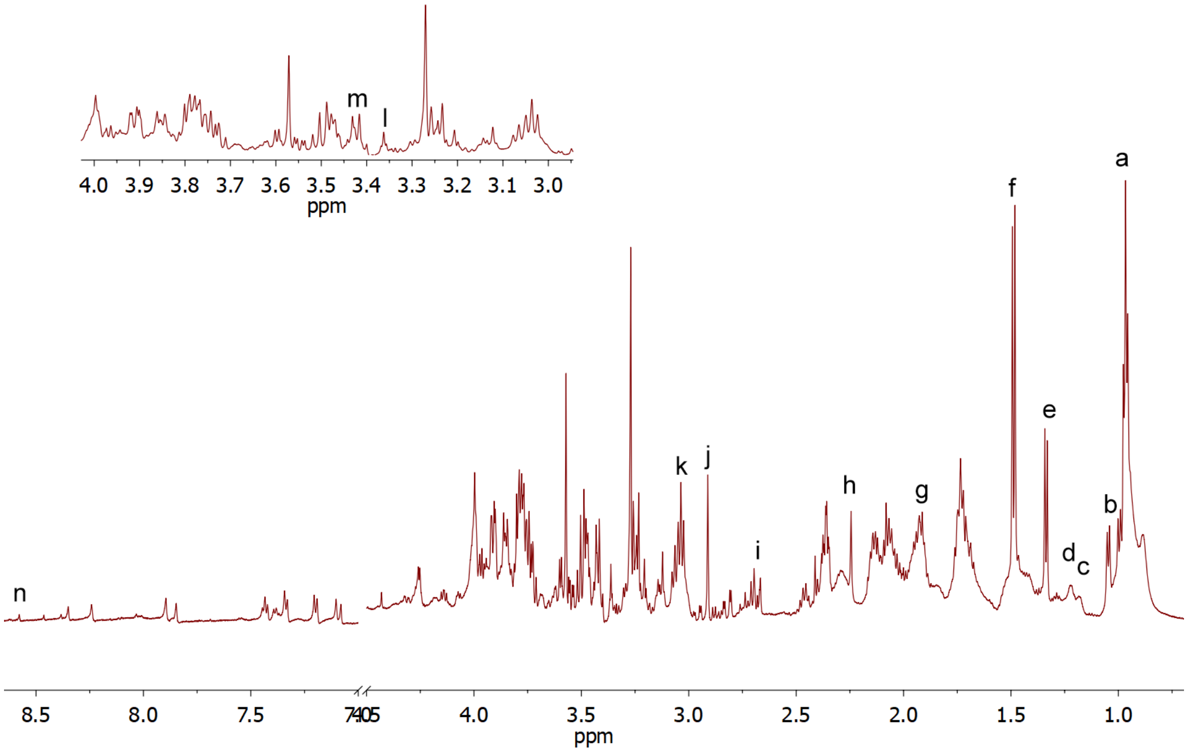

Figure S2. NMR spectrum from quality control group. a leucine, b valine, c 3-hydoxybutyrate, d methylmalonate, e lactate, f alanine, g acetate, h succinate, i citrate, j trimethylamine, k creatine, l taurine, m glycine, n phenylalanine.

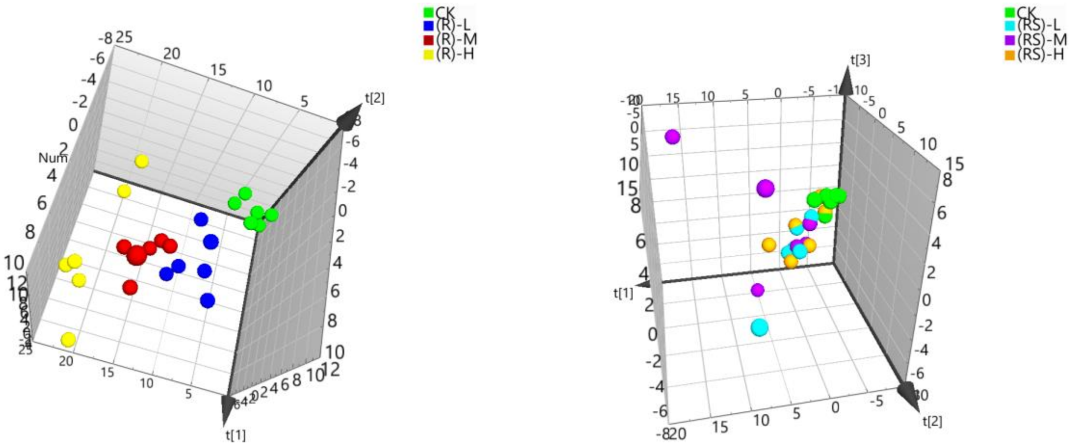

Figure S3. PCA of metalaxyl and metalaxyl-M at three concentration levels. RS-L: 0.5mg/kg metalaxyl, R-L: 0.5mg/kg metalaxyl-M. RS-M: 5.0mg/kg metalaxyl, R-M: 5.0mg/kg metalaxyl-M. RS-H: 50mg/kg metalaxyl, R-H: 50mg/kg metalaxyl-M.

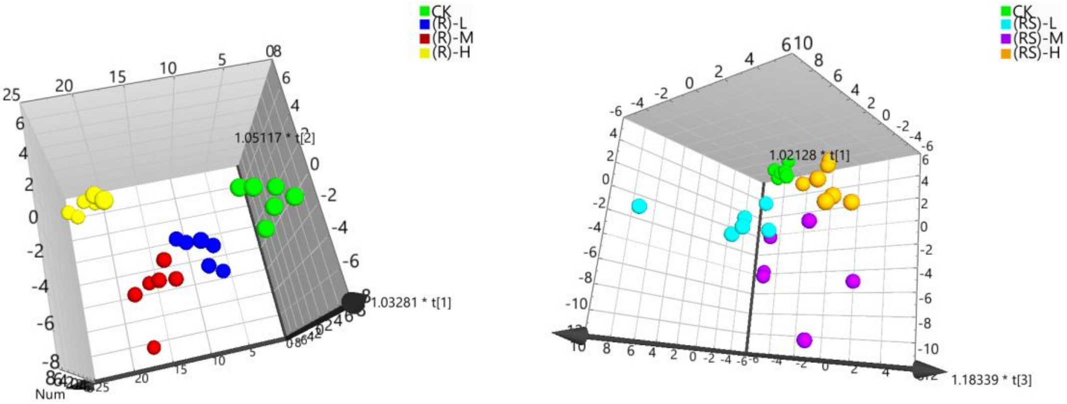

Figure S4. OPLS-DA (orthogonal partial least squares- discriminant analysis) of metalaxyl and metalaxyl-M at three concentration levels. RS-L: 0.5mg/kg metalaxyl, R-L: 0.5mg/kg metalaxyl-M. RS-M: 5.0mg/kg metalaxyl, R-M: 5.0mg/kg metalaxyl-M. RS-H: 50mg/kg metalaxyl, R-H: 50mg/kg metalaxyl-M.

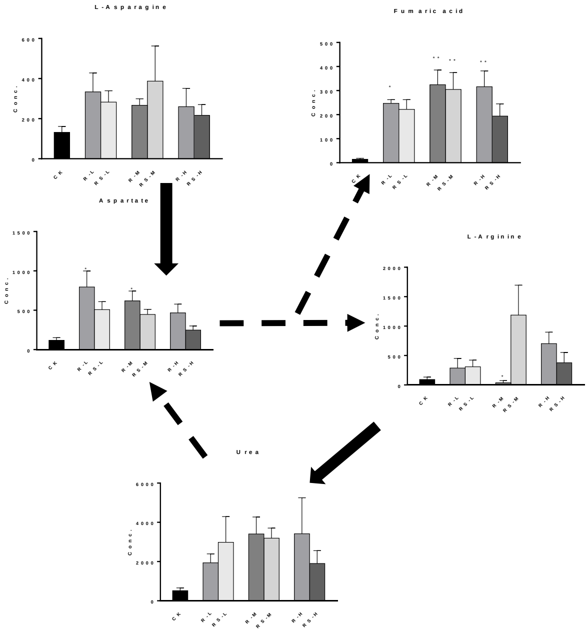

Figure S5. Disturbance of arginine biosynthesis metabolic pathway after metalaxyl and metalaxyl-M treatment. RS-L: 0.5mg/kg metalaxyl, R-L: 0.5mg/kg metalaxyl-M. RS-M: 5.0mg/kg metalaxyl, R-M: 5.0mg/kg metalaxyl-M. RS-H: 50mg/kg metalaxyl, R-H: 50mg/kg metalaxyl-M.

Table S1. The fold changes and VIPs of some metabolites after metalaxyl and metalaxyl-M exposed. RS-L: 0.5mg/kg metalaxyl, R-L: 0.5mg/kg metalaxyl-M. RS-M: 5.0mg/kg metalaxyl, R-M: 5.0mg/kg metalaxyl-M. RS-H: 50mg/kg metalaxyl, R-H: 50mg/kg metalaxyl-M.

No.	Compound	CK vs RS L		CK vs RS M		CK vs RS H		R L vs RS L		R M vs RS M		R H vs RS H	
		FC	VIP	FC	VIP	FC	VIP	FC	VIP	FC	VIP	FC	VIP
1	1-Methylhistidine	1.22	0.52	0.50	1.05	0.60	0.73	1.30	0.80	2.78	1.87	2.20	1.24
2	2-Hydroxybutyric acid	0.30	1.25	1.17	0.22	1.18	0.18	2.31	0.98	0.67	0.61	0.33	0.80
3	2-Hydroxyisovalerate	0.63	1.00	1.24	0.42	0.75	0.56	1.00	0.00	0.76	0.54	0.47	0.95
4	2-Oxopentanedioate	1.31	0.45	1.03	0.07	1.08	0.12	1.42	0.86	1.37	1.12	1.12	0.20

5	3-Hydroxybutyric acid	0.44	1.26	1.29	0.53	0.80	0.42	1.11	0.16	0.50	1.53	0.92	0.18
6	3-Hydroxyisobutyrate	1.57	0.72	1.34	0.42	0.65	0.45	0.34	1.47	0.00	1.83	0.69	0.27
7	3-Hydroxyisovaleric acid	126	1.20	0.10	1.47	83.4	1.02	1.23	0.28	529	1.82	0.03	0.92
8	3-Methylhistidine	0.68	0.44	0.31	1.06	2.25	0.96	0.93	0.09	27.4	1.76	0.58	0.66
9	5-Aminopentanoate	1.06	0.10	0.99	0.01	0.77	0.39	0.54	1.35	1.16	0.12	1.04	0.10
10	Acetic acid	1.31	0.61	1.25	0.37	1.70	1.15	1.17	0.35	1.61	0.99	0.46	1.56
11	Acetoacetate	0.96	0.05	1.09	0.26	1.03	0.04	0.47	1.20	0.88	0.36	0.77	0.54
12	Acetone	0.74	1.06	1.86	0.78	0.64	1.15	1.34	1.49	0.66	1.15	1.26	0.51
13	Alpha-ketoisovaleric acid	0.58	0.88	0.61	0.68	0.53	0.86	2.33	1.33	1.02	0.03	0.79	0.25
14	Ascorbic acid	0.92	0.13	1.14	0.37	0.19	1.44	1.89	0.89	31.0	1.35	4.46	1.12
15	Aspartate	1.68	1.33	1.22	0.36	0.93	0.21	1.29	1.05	1.36	0.40	1.13	0.43
16	Betaine	1.30	0.62	1.89	0.45	0.85	0.45	0.78	0.49	1.26	0.84	1.36	0.67
17	Butyrate	0.20	0.90	0.23	1.45	1.50	0.27	1.23	0.22	1.23	0.47	0.64	0.30
18	Carnitine	1.33	0.53	0.98	0.05	0.76	0.44	0.57	1.19	0.07	1.08	0.36	1.18
19	Choline	1.14	0.27	0.88	0.48	1.16	0.27	0.72	0.66	4.61	1.49	0.67	0.58
20	Citric acid	0.80	0.88	1.09	0.17	0.72	1.15	1.16	0.79	0.89	0.25	0.84	0.82
21	Creatine	1.33	0.65	0.51	0.63	0.43	1.21	0.41	1.77	0.82	0.99	0.22	1.28
22	Creatinine	2.45	1.27	0.01	1.15	2.89	1.03	0.44	1.57	0.46	1.41	0.13	1.28
23	D-Galactose	0.03	1.26	2.17	0.96	0.33	0.76	11.0	1.62	4.75	1.38	1.14	0.09
24	D-Glucose	1.07	0.10	1.15	0.13	2.33	1.12	1.46	0.77	0.71	0.61	1.97	1.42
25	Dimethyl sulfone	1.01	0.02	0.45	1.25	0.60	0.98	0.31	1.22	0.64	0.38	0.02	1.50
26	Dimethylamine	0.51	1.20	1.75	0.57	0.07	1.97	0.97	0.06	0.63	0.77	3.51	1.35
27	Dimethylglycine	1.38	0.39	0.55	0.85	0.08	1.07	0.86	0.21	0.96	0.07	5.06	0.68
28	Ethanol	0.73	0.49	3.71	1.13	0.52	0.88	0.82	0.38	0.47	1.25	1.29	0.47
29	Ethanolamine	1.52	0.55	0.51	1.51	1.81	0.56	2.30	1.34	0.86	0.19	1.12	0.13
30	Formate	0.60	1.68	0.12	0.87	0.43	1.95	1.16	0.88	0.89	0.33	0.65	1.33
31	Fructose	0.40	0.95	4.88	2.56	0.31	0.81	5.23	1.09	110	0.97	27.2	0.83
32	Fumaric acid	4.39	2.77	97.5	0.87	4.03	2.31	1.18	1.62	1.13	0.67	1.13	0.54
33	Glycerol	0.04	0.98	0.00	0.93	0.45	0.50	0.16	1.09	1158	1.18	0.12	0.78
34	Glycine	1.89	1.39	1.41	0.89	1.10	0.20	1.17	0.46	1.59	1.63	1.53	1.10
35	Glycolate	1.37	0.59	1.30	0.43	1.25	0.42	1.13	0.25	0.45	1.25	0.72	0.67
36	Guanidoacetate	2.77	0.61	0.03	0.87	2.57	0.50	0.91	0.08	23.9	1.44	0.69	0.24
37	Hippuric acid	1.41	1.19	0.97	0.09	1.37	1.07	0.76	1.60	0.85	1.02	0.62	1.74
38	Hydroxyphenyllactic acid	0.34	1.74	0.49	1.40	0.89	0.29	1.37	0.51	0.46	1.36	0.31	1.58
39	Hypoxanthine	0.66	1.73	0.61	1.69	0.82	0.87	1.01	0.14	1.00	0.01	0.75	1.08
40	Isobutyric acid	0.50	0.55	0.01	1.06	2.45	0.94	4.21	1.64	641	1.35	0.16	1.30
41	Isoleucine	1.31	0.55	0.85	0.24	1.25	0.40	0.55	1.23	1.49	0.78	0.52	0.94
42	Isopropyl alcohol	0.32	1.53	0.53	0.98	0.80	0.41	1.51	1.05	1.04	0.06	1.18	0.38
43	Isovaleric acid	0.34	1.77	0.71	0.65	0.52	1.28	1.11	0.19	0.44	1.22	0.73	0.77
44	L-Alanine	1.23	1.46	1.04	0.18	1.02	0.10	0.92	0.95	1.02	0.16	0.58	2.07
45	L-Arginine	0.99	0.03	2.28	1.09	1.98	0.86	0.85	0.35	0.04	1.97	1.05	0.08
46	L-Asparagine	0.49	1.47	0.42	1.38	0.26	1.78	1.32	0.44	1.07	0.11	2.16	1.07
47	L-Cysteine	0.37	1.24	0.41	1.17	0.52	0.87	2.23	1.26	0.78	0.38	1.15	0.18
48	L-Cystine	0.49	0.79	2.97	1.23	1.49	0.56	1.43	0.57	0.75	0.57	0.80	0.36
49	L-Fucose	0.01	1.13	0.83	0.13	0.39	0.64	60.5	1.62	0.11	0.87	0.01	1.05
50	L-Glutamic acid	2.04	1.86	1.16	0.30	1.80	1.42	1.02	0.16	1.52	1.32	1.01	0.05
51	L-Glutamine	1.10	0.11	0.47	0.83	0.88	0.17	0.47	0.76	2.50	0.95	0.22	1.01
52	L-Histidine	0.83	0.22	1.96	1.02	2.11	0.90	1.58	0.62	1.08	0.17	0.49	0.87
53	L-Lactic acid	1.84	1.30	0.79	0.39	0.49	0.92	0.93	0.25	2.28	1.47	0.57	0.46
54	L-Leucine	1.09	0.52	0.67	1.86	1.06	0.35	0.82	1.25	1.18	1.19	0.47	1.93
55	L-Lysine	1.31	0.50	0.88	0.22	2.38	1.53	0.80	0.49	1.43	0.81	0.74	0.71
56	L-Ornithine	0.87	0.10	1.37	0.23	0.00	0.90	0.38	0.67	0.12	1.08	21.6	0.85
57	L-Phenylalanine	1.57	0.99	1.18	0.26	2.18	1.17	0.66	1.06	0.66	0.68	0.84	0.30
58	L-Proline	1.01	0.01	1.21	0.29	1.01	0.02	1.12	0.24	0.98	0.04	0.69	0.45
59	L-Serine	1.21	0.42	1.80	0.87	1.66	1.07	1.07	0.12	0.52	1.05	2.01	2.03
60	L-Threonine	0.83	0.63	1.28	1.10	1.23	0.93	1.09	0.33	0.72	1.70	0.95	0.24
61	Malonate	1.03	0.05	0.41	1.85	0.48	1.16	0.43	0.97	1.38	0.49	1.44	0.42
62	Mannose	0.26	0.72	0.54	0.37	0.02	0.90	0.13	1.09	2.23	0.51	92.6	1.16
63	Methanol	0.63	1.07	0.75	0.56	1.00	0.01	1.45	0.95	0.96	0.08	2.45	1.20
64	Methionine	0.63	0.92	0.48	1.21	0.66	0.73	0.74	0.50	1.36	0.50	0.94	0.09
65	Methylamine	0.80	0.87	0.59	1.56	0.33	2.49	0.98	0.07	1.20	0.61	1.52	1.53
66	Methylmalonic acid	0.91	0.18	0.72	0.46	0.45	1.07	1.12	0.17	0.82	0.29	1.66	0.70

67	Myo-inositol	0.14	1.24	0.34	0.80	0.00	1.33	10.5	1.48	0.38	0.58	54.3	0.83
68	Oxalacetic acid	0.77	0.25	1.63	0.60	1.27	0.34	1.41	0.32	0.61	0.78	0.03	1.60
69	p-Cresol sulfate	1.10	0.52	1.30	1.09	1.54	1.41	1.29	1.78	0.87	0.54	0.56	1.53
70	Phenylacetate	1.06	0.38	0.67	1.74	1.21	0.98	0.81	1.22	1.24	1.32	0.32	2.23
71	Phosphorylcholine	0.91	0.17	1.13	0.24	1.17	0.33	1.79	1.20	1.21	0.36	1.04	0.08
72	p-Hydroxyphenylacetic acid	1.00	0.01	0.56	1.45	0.81	0.67	0.78	1.36	1.35	1.32	0.60	1.47
73	Propylene glycol	1.71	0.66	0.72	0.32	1.21	0.23	0.57	0.88	0.09	1.24	1.05	0.08
74	Pyroglutamic acid	0.46	0.52	0.19	0.71	0.94	0.04	6.77	1.13	2.44	0.56	0.67	0.32
75	Pyruvic acid	2.03	1.00	1.83	0.82	0.59	0.60	0.67	0.76	0.90	0.17	1.36	0.41
76	Sarcosine	0.69	1.05	0.43	1.75	0.02	2.54	0.85	0.43	0.59	0.89	10.25	2.11
77	Sorbitol	6.88	1.21	0.43	0.87	39.2	0.76	5.25	1.07	3.97	0.97	2.88	0.58
78	Succinate	0.03	1.68	0.17	1.31	0.12	1.46	6.23	2.49	0.19	0.91	0.20	0.91
79	Taurine	3.98	1.12	6.16	1.79	1.03	0.03	0.65	0.55	0.67	0.80	1.58	0.41
80	Trimethylamine	0.57	0.86	1.87	1.25	1.12	0.20	2.78	2.00	0.47	1.84	1.45	0.93
81	Tryptophan	0.13	2.22	0.12	2.00	0.72	0.84	2.94	1.35	2.49	1.06	0.52	1.42
82	Tyrosine	2.66	1.29	1.55	0.56	2.89	1.42	0.46	1.38	0.54	1.07	0.63	0.74
83	Uracil	0.86	0.39	1.27	0.61	0.31	1.70	0.93	0.21	0.63	1.23	0.89	0.15
84	Urea	0.64	0.87	1.15	0.34	0.74	0.57	0.89	0.46	0.82	0.73	1.79	0.95
85	Valine	0.83	0.14	0.21	0.80	0.92	0.06	0.57	0.43	1.14	0.15	0.75	0.23
86	Xanthine	0.69	1.26	0.37	1.98	0.63	1.29	0.94	0.32	1.29	0.61	0.53	1.44
